# Letter to the Editor: Medical Students Improving Health Care Beyond Clinical Rotations During the COVID-19 Outbreak

**DOI:** 10.1177/1062860620931934

**Published:** 2020-05-31

**Authors:** Don Hayes

To the Editor:

Due to the COVID-19 outbreak, the Association of American Medical Colleges (AAMC) recommended the suspension of all direct patient contact for clinical rotations by medical students effective immediately on March 17, 2020.^1^ The reasons for the decision were multifactorial, including the opportunity for medical schools to develop and implement educational programs to fully educate all their students, to ensure medical student and patient safety, and to conserve personal protective equipment.

Apprenticeship on clinical rotations remains as relevant today in the education of medical students as it did when Sir William Osler, FRS, FRCP, established clerkships a century ago, giving students a role on clinical services.^2^ Endorsed by the AAMC, Abraham Flexner later influenced medical education through recommendations of science-based programs in university departments linked to teaching hospitals.^2^ With COVID-19 being the first pandemic to affect the United States in the modern era, the outbreak resulted in the first immediate cessation of clinical education for medical students in the United States.

With no precedent, medical students with and without faculty oversight across the country acted through the development of both social connectedness and wellness, but more importantly community service, to assist patients in the general population. Locally, medical students at the University of Cincinnati College of Medicine developed a free service program through which student volunteers provided free delivery of groceries, medications, and meals to patients at risk for COVID-19-associated complications if they acquire the viral infection. Moreover, a group of medical students at Harvard Medical School (HMS) implemented a team of 500 volunteers to develop a student-led organization to optimize mobilization of interested students in the COVID-19 response, both clinically and in the community, and to serve as a liaison between administration for the needs and efforts of these engaged students.^3^ The HMS organizational framework was shared with multiple medical schools across the United States.

Our job as physicians is to heal the sick, especially when patients are at their most vulnerable. The innate characteristics of physicians to heal patients is developed before graduation from medical school. Many students develop optimistic and altruistic behavior and a sense of morality patients during their medical education.^4^ During this unprecedented time when medical students are removed from clinical services at US teaching hospitals, they do not have to feel powerless as noted by the HMS students. Medical students can empower themselves through sustaining the quality of health of the most vulnerable in the broader community.

Don Hayes Jr, MD, MS, MEd *University of Cincinnati, Cincinnati,
OH* *Cincinnati Children’s Hospital Medical Center, Cincinnati,
OH*
